# Recent Advances in the Genetics of Fractures in Osteoporosis

**DOI:** 10.3389/fendo.2019.00337

**Published:** 2019-06-04

**Authors:** Fjorda Koromani, Katerina Trajanoska, Fernando Rivadeneira, Ling Oei

**Affiliations:** ^1^Department of Internal Medicine, Erasmus MC University Medical Center Rotterdam, Rotterdam, Netherlands; ^2^Department of Epidemiology, Erasmus MC University Medical Center Rotterdam, Rotterdam, Netherlands; ^3^Department of Radiology and Nuclear Medicine, Erasmus MC University Medical Center Rotterdam, Rotterdam, Netherlands

**Keywords:** genetics, osteoporosis, fracture risk, genome-wide association studies, review, family, single nucleotide polymorphism, copy number variation

## Abstract

Genetic susceptibility, together with old age, female sex, and low bone mineral density (BMD) are amongst the strongest determinants of fracture risk. Tmost recent large-scale genome-wide association study (GWAS) meta-analysis has yielded fifteen loci. This review focuses on the advances in the research of genetic determinants of fracture risk. We first discuss the genetic architecture of fracture risk, touching upon different methods and overall findings. We then discuss in a second paragraph the most recent advances in the field and focus on the genetics of fracture risk and also of other endophenotypes closely related to fracture risk such as bone mineral density (BMD). Application of state-of-the-art methodology such as Mendelian randzation in fracture GWAS are reviewed. The final part of this review touches upon potential future directions in genetic research of osteoporotic fractures.

## Genetic Architecture of Fracture Risk

Bone fractures are considered the most relevant clinical sequelae of osteoporosis. Genetic susceptibility, together with old age, female sex, falls (1) and low BMD are amongst the strongest determinants of fracture risk. A positive family history is a risk factor for osteoporosis and fractures thus reinforcing the role of genetics in the basis of liability to osteoporotic fractures (2). Moreover, parental hip fracture has been incorporated as a risk factor in the FRAX clinical assessment algorithm in the last decade. Heritability studies have reported estimates for bone mineral density (BMD) and fractures of up to 66 and 46%, respectively (3, 4). A parental history of fracture has been related to any-type of fracture risk (risk ratio (RR) for any-type of fracture 1.17, 95% CI 1.07-1.21), and hip fracture (RR 1.49, 95% CI: 1.17-1.89) (5). These previous findings are at the background of further genetic investigations.

Different types of genetic changes may underlie diseases; structural variations, including deletions or base pair changes, vary from mutations of larger stretches of genetic material to single nucide polymorphisms (SNPs) and mutations affecting 1 base pair together with structural variation comprising insertions and deletions of different size across the genome. As discussed elsewhere in this journal issue, there are a multitude of genetic mutations known to cause relatively infrequent monogenic conditions presenting with bone fragility including familial forms of osteoporosis, osteogenesis imperfecta and other bone disorders, for example: *COL1A1* (6), *COL1A2, LRP5* (7), *WNT1* (8), *LGR4* (9), *PLS3* (10), *CRTAP, FKBP10, LEPRE1, PLOD2, PPIB, SERPINF1, SERPINH1* and *SP7* (11), summarized in Table 1. One human genome contains roughly 3 billion (3,000,000,000) nucleotides, which are the building blocks of the genome in the form of the letters A, T, G, and C. When a SNP in the sequence is swapped for another letter, this is called a mutation and considered a SNP when occurring relatively frequent, i.e., with a minor allele frequency (MAF) >0.5% in the population). Technologies for SNP genotyping include enzyme-based methods (e.g., polymerase chain reaction [PCR]-based), hybridization-based methods (e.g., microarrays) and next-generation sequencing.

**Table 1:** An overview of monogenic bone disorders and the genes involved in their pathology.

**Disease**	**Gene**	**Locus**	**References**
Autosomal dominant Osteopetrosis type II	*CLCN7*	16p13	(12)
Autosomal dominant hypophosphataemic rickets	*FGF23*	12p13.32	(13)
Early-onset osteoporosis	*WNT1*	12q13.12	(8)
Familial hypocalciuric hypercalcaemia (FHH)	*CASR*	3q21.1	(14)
	*GNA11*	19p13.3	(15)
	*AP2S1*	19q13.3	(16)
Hereditary hypophosphataemic rickets with hypercalciuria	*SLC34A3*	9q34.3	(17)
Hypophosphatasia	*TNS/ALPL*	1p36.12	(18)
Juvenile Paget disease	*TNFRSF11B*	8q24.12	(19)
Osteogenesis imperfecta (OI)	*COL1A1*	17q21.33	(6)
	*COL1A2*	7q21.3	(7)
	*IFITM5*	11p15.5	(20)
	*SERPINF1*	17p13.3	(11)
	*CRTAP*	3p22.3	(11)
	*PRH1/LEPRE1*	1p34.2	(11)
	*WNT1*	12q13.12	(8)
Pseudohypoparathyroidism	*GNAS*	20q13.3	(21)
Sclerostosis	*SOST*	17q21.31	(22)
	*LRP4*	11p11.2	(23)
Vitamin D-dependent rickets	*CYP3A4*	7q22.1	(24)
	*CYP27B1*	12q14.1	(24)
	*VDR*	12q13.11	(25)
X-linked hypophosphatemic (XLH) rickets	*PHEX*	Xp22.11	(11)
X-linked osteoporosis	*PLS3*	Xq23	(11)

Genome-wide screening, as applied in genome-wide association studies (GWAS), tests for associations between genetic markers (SNPs and traits of interests in a hypothesis -free manner. This approach can add onto *a priori* knowledge about the physiological, biochemical or functional aspects of possible candidates (26). On the other hand, genome-wide genotyping is unbiased in the sense that by surveying the whole genome in a hypothesis-free manner, involvement of unexpected candidates or even loci with unknown function could be revealed (27). Meta-analyses are an appropriate way for follow-up in candidate gene studies of top loci and genes prioritized by GWAS, and use of existent GWAS for look-ups of functional biological hypotheses.

It has been shown that SNPs underlie differences between people, including the variability in disease susceptibility, and recent GWAS have vastly expanded our knowledge in this area (28). Apart from developing our understanding of disease etiology, expectations are that these genetic markers will be useful in disease diagnostics and prediction, form potential drug targets and potentially modulate treatment response (9).

Fracture is the most clinically relevant endpoint of osteoporosis and its etiology is complex. Similarly to other traits strongly related with old age, the heritability of fracture risk decreases with age. Studying correlated endophenotypes that are associated with fracture risk, such as BMD, lean mass and hand grip strength might be a good alternative to study the genetic basis of fracture risk. GWAS for various osteoporosis-related traits have shown that targeting these quantitative endophenotypes with excellent measurement properties (root mean square standard deviation expressed as coefficient of variation of 1.0–1.2% for the spine and 1.1–2.2% for the femoral neck by DXA)(29) is efficient in the number of loci discovered. The earliest GWAS of DXA-BMD identified 24 loci that influence DXA-BMD variation explaining ~3% of trait variance (30–36) of which several variants have also been nominally associated with fracture risk (37, 38). A breakthrough was the meta-analysis by the genetic factors for osteoporosis (GEFOS) and genetic markers for osteoporosis (GENOMOS) consortia (39), where the top-associated BMD markers explaining ~6% of BMD variance were also tested for fracture risk (31,016 cases and 102,444 controls), where 14 out of 56 BMD loci were associated at Bonferroni corrected significance level with fractures, of which six loci at genome-wide significant level. An alternative measurement method for DXA is total body BMD, as is more commonly applied in childhood and adolescence, where GWAS recently reported more than 80 loci explaining 10% of the variance (40). This same publication examined these SNPs in an independent fracture study, where a decrease of one standard deviation in genetically determined total body BMD resulted in 56% higher odds of fracture. Another endophenotype is BMD estimated from quantitative heel ultrasound, where in this GWAS 12 out of the associated 307 SNPs were also associated with fracture risk, newly adding the *AQP1* and *SLC8A1* loci as potential fracture genetic determinants (41).

BMD is among the quantitative traits for which GWAS have been effective in discovering high numbers of loci (42, 43). On the other end, GWAS for dichotomous disease as a direct outcome have yielded relatively lower numbers of loci discovered (42), probably due to study power issues. This might concern the studies for osteoporotic fractures as well. Further, identifying the specific genetic determinants contributing to the risk of fracture has been difficult due to its multifactorial nature and occurrence late in life. High phenotype heterogeneity and ascertainment bias reduce the power to detect association, making the genetic studies even more difficult. Endophenotypes may be nearer to the coding DNA in the chain of events at the basis of multifactorial diseases, and, homogeneous determination of endophenotypes may be simpler than defining certain diseases. Indeed, hypothesis-free genome-wide screens have shown that the most prominent and consistently replicating genetic loci associated with fracture risk are also associated with BMD, which serves as proof of BMD being a very powerful endophenotype for fracture prediction (44). This also implies that an underlying fragility component mediated through genetic predisposition seems to form a major part of the basis for fracture risk.

At the beginning of the GWAS era, the genomics field was dominated by the common disease-common variant hypothesis, which states that common diseases are caused by common genetic variants (45). Yet, the list of rare genetic variants influencing common disease is growing (46). In between these two categories are SNPs with minor allele frequency (MAF) of 0.5–5%.

## Recent Advances in the Genetics of Osteoporotic Fractures

**Figure 1:**
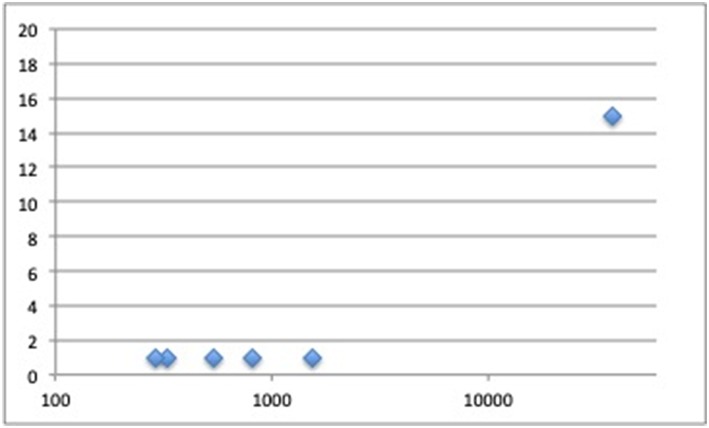
Number of loci discovered in fracture genome-wide association studies (Y-axis) plotted by fracture cases sample size (X-axis).

Several GWAS specifically aimed at fracture risk, have been performed to date, as discussed below and summarized in Table 2 and Figure 1.

Table 2Findings of fracture risk genome wide association studies.

**Table:** 

Sample size fracture cases vs. controls	Type of fracture	Ethnicity	Type of genetic variation	References
**A. PUBLISHED FRACTURE RISK GENOME WIDE ASSOCIATION STUDIES**				
329 vs. 2,666	Vertebral (radiographic)	Caucasian	Single nucleotide polymorphism	Oei et al. (47)
288 vs. 1,139	Any	Asian	Single nucleotide polymorphism	Hwang et al. (48)
809 vs. 4,369	Any	Caucasian	Copy number variation	Oei et al. (49)
540 vs. 10,305	Any	African-American	Single nucleotide polymorphism	Taylor et al. (50)
1,553 vs. 4,340	Vertebral (clinical)	Caucasian	Single nucleotide polymorphism	Alonso et al. (51)
37,857 vs. 227,116	Any	Caucasian	Single nucleotide polymorphism	Trajanoska et al. (44)

**Table:** 

References	Variant	Effect allele	Effect allele frequency	Alternate allele	Odds ratio	95% Confidence interval	Locus	Candidate gene
**B. GENETIC VARIANTS FOUND ASSOCIATED IN THE FRACTURE RISK GENOME WIDE ASSOCIATION STUDIES**								
Oei et al. (47)	rs11645938	C	9.65%	T	1.06	0.98–1.14	6p25.1	*FOXC2*
Hwang et al. (48)	rs784288	A	25%	G	1.39	1.24–1.56	3q26.2	*MECOM*
Oei et al. (49)	210 kb deletion	N.A.	0.14%	N.A.	3.11	1.01–8.22	6p25.1	*PECI*
Taylor et al. (50)	rs12775980	A	3%	C	2.12	1.61–2.79	10p11.23	*SVIL*
Alonso et al. (51)	rs10190845	A	4.9%	C	1.74	1.06–2.06	2q13	*FBLN7*
Trajanoska et al. (44)	rs4233949	G	61%	C	1.03	1.02–−1.04	2p16.2	*SPTBN1*
	rs430727	T	45%	C	1.03	1.02–1.04	3p22.1	*CTNNB1*
	rs10457487	C	51%	A	1.05	1.04–1.06	6q22.33	*RSPO3*
	rs2982570	C	58%	T	1.04	1.03–1.05	6q25.1	*ESR1*
	rs2908007	A	60%	G	1.06	1.05–1.07	7q31.31	*WNT16*
	rs6465508	G	34%	A	1.04	1.03–1.05	7q21.3	*C7orf76*
	rs6959212	T	34%	C	1.03	1.02–1.04	7p14.1	*STARD3NL*
	rs1548607	G	32%	A	1.03	1.02–1.05	7p12.1	*GRB10*
	rs7851693	G	35%	C	1.04	1.03–1.05	9q34.11	*FUBP3*
	rs11003047	G	11%	T	1.09	1.07–1.10	10q21.1	*MBL2*
	rs3736228	T	15%	C	1.06	1.05–1.08	11q13.2	*LRP5*
	rs1286083	T	82%	C	1.05	1.04–1.07	14q32.11	*RPS6KA5*
	rs2741856	G	92%	C	1.10	1.07–1.11	17q21.31	*SOST*
	rs4635400	A	36%	G	1.04	1.03–1.05	18p11.21	*FAM210A*
	rs9980072	G	73%	A	1.04	1.03–1.05	21q22.2	*ETS2*

### GWAS for Fracture Risk and DXA-BMD

With regard to the allele frequencies, osteoporotic fracture risk has been shown to be associated with common, uncommon and rare variants. In a study of structural variation in relation to fracture risk (5,178 Dutch individuals of which 809 fracture cases), the proportion of fracture cases with at least one deletion was significantly higher compared to controls and a 210 kb deletion located on chromosome 6p25.1 was associated with fracture risk (OR = 32.58, 95% CI 3.95 to 1488.89). An *in silico* meta-analysis in four studies with copy number variation microarray data found similar results for the association with fracture risk (OR 3.11, 95% CI 1.01 to 8.22). Notably, this variant was absent in samples from several countries; indicating geographic diversity.

Nevertheless, this study indicates that the study of rare CNVs deserves follow-up (49). Also, another effort in the GEFOS and GENOMOS consortium encompassing for the first time a sequencing-based GWAS meta-analysis has discovered *EN1* as a determinant of bone density and fracture (rs11692564(C) allele OR = 1.18) (52). Further, deCODE investigators have discovered common sequence variants in *PTCH1* (53) (MAF = 11.4–22.6%) and less frequent (MAF = 0.14%−0.18%) variants in *LGR4* (9) associated with BMD and fractures (OR = 1.09 and OR = 3.12).

The first two published GWAS for fracture risk identified the *SVIL* gene locus in African American populations (50) and the MECOM gene locus in Korean and Japanese populations (48), respectively. It should however be noted that access to larger sample sizes is still limited for samples of non-European descent, as reflected in a lack of a replication meta-analysis for the African American fracture GWAS. The second GEFOS GWAS meta-analysis for BMD assessed the identified loci for their relation with fracture (39). The recently published large scale GWAS meta-analysis for fracture in 25 cohorts from all over the world with genome wide genotyping and fracture data (discovery in 37,857 fracture cases and 227,116 controls; replication in up to 147,200 fracture cases and 150,085 controls) identified 15 loci (44), of which all were also associated with bone mineral density. Relative to the previous DXA-BMD GWAS–fracture association study (39), we confirmed the 2p16.2 (*SPTBN1*), 7q21.3 (*SHFM1*), 10q21.1 (*MBL2*/*DKK1*), 11q13.2 (*LRP5*), and 18p11.21 (*FAM210A*) loci, and observed an increased signal at *SOST, CPED1/WNT16, FUPB3, DCDC5, RPS6KA5, STARD3NL*, and *CTNNB1*. Additionally, we added the 6q22.33 (*RSPO3*), 6q25.1 (*ESR1*), 7p12.1 (*GRB10*/*COBL*), and 21q22.2 (*ETS2*) loci to the list of novel fracture loci. The signals mapped to genes clustering in pathways known to be critical to bone biology (e.g., *SOST, WNT16*, and *ESR1*) or novel pathways (*FAM210A, GRB10*, and *ETS2*). These variants explain approximately 2% of variance in fracture risk (unpublished data).

As reviewed elsewhere (54), several Mendelian randomization (MR) studies in relation to fracture risk have been published. One of the first publications in this field was an exploration of the association between C-reactive protein levels and increased fracture risk, where we did not find evidence for a causal effect (55). Nevertheless, particularly for proving negative associations well-powered meta-analyses are required. The largest MR study to date was conducted on behalf of the GEFOS/GENOMOS consortium and the 23andMe research teams (44). In this study, SNPs that had been previously reported in GWAS were used as instrumental variables, representing 15 risk factors for fracture including: BMD (femoral neck and lumbar spine), age of puberty, age at menopause, grip strength, vitamin D, homocysteine, thyroid stimulating hormone level, fasting glucose, type 1 diabetes, type 2 diabetes, rheumatoid arthritis, inflammatory bowel disease, coronary artery disease, and the lactose intolerance marker (rs4988235) as a surrogate to assess long term differences in dairy derived calcium intake. SNPs influencing BMD were strongly and inversely correlated with odds of fracture (for femoral neck BMD SNPs genetic correlation −0.59; and for lumbar spine BMD SNPs genetic correlation −0.53). By contrast, of the remaining clinical risk factors evaluated, only homocysteine was shown to be genetically correlated with fracture risk (genetic correlation >0.2 or <-0.2, and surpassing the threshold for statistical significance for multiple testing), but this should be interpreted with caution as the confidence interval is wide. In the subsequent Mendelian randomization analysis, again, only the BMD SNPs were significantly associated with fracture risk. This implies a causal effect of these SNPs through BMD on fracture risk, without any evidence for pleiotropic effects as the Mendelian randomization-Egger regression intercepts centered around zero. By contrast, despite high statistical power, none of the other tested and well-accepted risk factors had evidence for a major causal effect on fracture risk. These results should be interpreted with caution as reviewed elsewhere (56). Still study power is limited in spite of the large sample sizes and the LD score regression method used. Potentially existing pleiotropy or non-linear relationships (e.g., threshold effects and extremes of the population) may be subjects of future research. Another very recent study (57) extensively assessed genetic determinants of osteoporosis, combining the UK Biobank and 23andMe cohorts (57). The authors, first identified 518 genome-wide significant loci (of which 301 novel) associated with heel BMD and then identified 13 loci associated with fractures across 1.2 million individuals (all also associated with heel BMD). Furthermore, they identified target genes known to influence bone density and strength and performed a rapid throughput skeletal phenotyping of 126 knockout mice with disruptions in predicted target genes. They found an increased abnormal skeletal phenotype frequency compared to unselected lines and a further in depth analysis on gene DAAM2 showed a disproportionate decrease in bone strength relative to mineralization.

Another Mendelian randomization study is the report on a causal effect of serum estradiol concentrations (interestingly in men) and an increased risk of any fracture (OR 1.35, 95% CI, 1.18-1.55), non-vertebral major osteoporotic fractures (OR 1.75, 95% CI, 1.35-2.27) and wrist fractures (OR 2.27, 95% CI, 1.62-3.16) (58).

Although most genetic studies on fracture risk have pulled together fracture information of any type, without discrimination of site, there are two major efforts on vertebral fracture GWAS that have been published. The first genome-wide association study for radiographic vertebral fractures in the Rotterdam Study, found a marker on chromosome 16q24 as genome-wide significantly associated (59). Although the 16q24 locus was found associated with BMD and vertebral defects at birth before, the association with vertebral fracture risk could not be replicated by *de-novo* genotyping across 15 studies worldwide, likely due to the heterogeneity underlying the different fracture definitions. A subsequent publication focusing on clinical vertebral fractures (i.e., those presenting with clinical manifestations) identified and replicated a locus tagged by rs10190845 on chromosome 2q13 where differential expression of the positional candidate genes TTL and SLC20A1 was shown (51).

### Recent GWAS for Heel BMD and Other Endophenotypes

The most recent study by Morris et al. (57) identified 518 genome wide significant loci (of which 301 novel) across 426,824 individuals of UK-Biobank which altogether explain around 20% in heel BMD variance. Earlier in 2017, Kemp et al. had identified across a subsample of UK-Biobank (*N* = 142,487) 203 loci, of which 153 novel at the time of publication (41).

Lean mass and hand grip strength have been associated with fracture risk (60) and may provide a possible endophenotype for potential genetic studies to elucidate fracture risk. It is thought that this relationship may be because of an inverse relationship between muscle strength and balance and thus fall risk. A study by Zillikens et al. (61) found five SNPs in/near HSD17B11, VCAN, ADAMTSL3, IRS1, and FTO for total body lean mass across 101767 individuals and three SNPs in/near VCAN, ADAMTSL3, and IRS1 for appendicular lean mass among 73,420 individuals. Karasik et al. (62) additionally identified a novel LM locus (TNRC6B).

Hand grip strength GWAS by Willems et al. was associated with 16 new loci. Furthermore, in the same study, the authors found evidence of shared genetic etiology of BMD and lean mass with grip strength and moreover a suggestive causal role for higher grip strength and lower risk of fracture (63). Similar results were found for the potential causal relationship between hand grip strength and fracture risk, but could not be replicated with a multiple testing significance threshold in the study by Trajanoska et al. (44).

## Potential Future Directions in Genetic Research of Osteoporotic Fractures

### Increasing Sample Size

A minimum sample-size threshold needs to be reached in GWAS, from where the number of discovered loci increases along with growing sample sizes as study power improves (42). Mega-sized biobanks, such as 23andMe and UK Biobank, including hundreds of thousands of participants with GWAS are increasingly becoming available (64, 65). A drawback from such Mega-GWAS is that phenotype data tends to be of variable quality and less accurate. However, there is a trade-off where the huge numbers may boost study power tremendously and overcome measurement error to a certain extent. In addition, the success rate of unraveling underlying genetic mechanisms may be influenced by the complexity of the genetic architecture of the trait of interest, including imperfect penetrance, allelic heterogeneity, and gene-environment and epigenetic effects (42, 43). The discovery of rare variants is hindered by the large sample sizes required to attain sufficient study power, where research consortia and Mega-GWAS with even larger sample sizes prove their worth through ever-increasing sized meta-analyses. Larger imputation reference panels and sequencing-based genotyping are becoming progressively available, facilitating more accurate examination of lower-frequency SNPs and other type of genetic variants such as indels and larger deletions (66).

Furthermore, it has been proposed that the missing heritability for human height and body mass index is likely to be small after estimating the genetic variance from all imputed variants (67); this will likely be the case for a (quantitative) trait such as BMD as well. Until now, rare variant association studies have found variants with larger effects where each explains only a tiny proportion of the phenotypic variance, because the heritability explained is dependent on the effect size and allele frequency (68). Therefore, arguments can be found to study both common and rare variants in the occurrence of common diseases (68), as also confirmed by our experiences in the bone field.

### Increasing Phenotyping Quality

More detailed phenotyping is believed to be of value for scrutinizing skeletal-site specific effects for fracture risk, for example cortical vs. trabecular bone, which justifies separate GWAS efforts for specific fracture types. This thinking comes from the observations that heritability of BMD varies across skeletal sites due to a mixture of shared and specific genetic and environmental influences as quantified by the genetic correlations (69), which supports the findings that some genetic loci display skeletal-site specific effects (32). Furthermore, it has been hypothesized that using stricter phenotype definitions and taking into account fracture mechanisms may increase study power. Yet, a major drawback is the decreasing sample size. Results for radiographic (59) and clinical vertebral fractures (51) have been published, as described above, efforts for hip and wrist fractures are underway, but struggle with attaining sufficient study samples to enable discoveries. Therefore, the all-type of fracture GWAS approach seems the starting point to attain maximum sample size for power to perform the first screening for genetic variants that contribute to osteoporotic fracture risk in general. Other even more specific subjects of clinical studies could be atypical (femoral) fractures or fracture healing, which could yield insight into differences in natural healing mechanisms and efficacy of medical treatment between patients.

The tough start of the fracture GWAS may be rooted in the complex phenotype definition and heterogeneity of the trait and its underlying genetics. A better understanding of the genetic architecture seems necessary. More clarity is needed which fracture phenotypes should be studied together because they have a joint genetic etiology, and which do not and thus should be analyzed separately; for example vertebral vs. non-vertebral fractures are distinguished clinically and probably also genetically. Then robust selection criteria should be defined for an optimal fracture phenotype definition of interest. Research ideas include data enrichment for cases that have a known family history for osteoporosis, having fractured at relatively young age or having sustained multiple fractures. This because the heritability of osteoporotic fractures at younger age is higher (4). Nonetheless, the osteoporotic fracture incidence at young age is lower, which may limit study sample sizes. Theoretically, it has been speculated that perhaps further exclusion criteria need to be established for cases that are thought to be caused by arguably non-genetic mechanisms (e.g., non-genetic secondary osteoporosis, high-trauma, old age, malnourishment, etc.), where refinement and automatization of measurements may enhance the richness, quality and quantity of research data available. However, until now in practice, bigger seems better to efficiently identify genes; then one should take these discoveries and bring them in a candidate-gene context and look across rich sets of detailed phenotypes that help understand the underlying biology. Combination into multivariate GWAS of multiple disease-related traits could further exploit the detection of pleiotropic effects (70) and novel statistical methods may be able to better utilize the richer phenotype information that will become available (71, 72).

Additionally, richer phenotyping of endophenotypes may yield more insight. Dual energy X-ray absorptiometry still misses 80% of patients who will fracture (73). One of the underlying reasons is that it generates two-dimensional scans and does not sufficiently appreciate bone microarchitecture, an important determinant of bone strength (74). Areal BMD does appreciate bone size and in part the internal architecture; the trabecular bone score (TBS) which can also be derived from DXA data will be worth further investigations (75). Further improvements require more advanced imaging than dual energy X-ray absorptiometry, principally by direct three-dimensional radiological imaging investigations, such as computed tomography or magnetic resonance imaging, to directly visualize microstructure, differentiate cortical and trabecular bone, and model bone strength biomechanically (76). Second, the contribution of the mineral phase to bone's mechanical properties has dominated scientific thinking, while bone is composed of three different phases (by volume: mineral 42%, collagen matrix 35%, and water 23%) (77). Novel imaging techniques that can quantify this bone composition are coming up (78), and genetic studies into these endophenotypes are yet to come.

Finally, it could be argued that bone geometry and its genetics should be studied. Intriguingly, taller persons are at increased risk of fractures in spite of having larger bones with more mass (79, 80). This may be caused by a different distribution of bone mass by periosteal apposition (81). Further, loci implicated in the GWAS of human stature are enriched for genes important for skeletal growth (82). And more specifically, a GWAS meta-analysis for hip shape was published very recently and found 17q24.3 and ASTN2 as associated in lookups in hip fracture GWAS (unpublished data) (83).

### Richer Genotyping

However, some of the measurement methods with respect to both genotyping and phenotyping currently available are simply too expensive or invasive to apply on a population level at present. Yet, current limits are being challenged, with the very first successful large-scale applications of whole-genome sequencing and deep imputation using sequencing-based reference panels in the osteoporosis research field (52). The Haplotype Reference Consortium (HRC) and the Trans-Omics for Precision Medicine (TOPMed) Program have created large reference panels of human haplotypes by combining together sequencing data from multiple cohorts. Further studies of copy number and structural variations should be performed. However, the genome may be too distant in the cascade from the disease of interest to detect clinically relevant patterns, therefore, screening the transcriptome, epigenome, metabolome, proteome and even microbiome at perhaps multiple time points may prove necessary. This may be applied to clinical fracture patient studies as well as population-based cohorts, where subgroups could be studied including for example individuals with multiple fractures, persons with fractures at young age, and elderly individuals free of fractures. The osteoporosis field has started to explore epigenetic regulation for instance: microRNA (84, 85), long non-coding RNA (86), gene expression (87), and DNA methylation (88).

### Functional Follow-Up

Oftentimes the function of genes contained in the associated loci are not (completely) known. Functional follow-up studies are needed, yet, the development of animal knock-out-models may take years. Establishment of multi-disciplinary research consortia worldwide may be beneficial to efficiently take GWAS discoveries to functional follow-up in a harmonized research pipeline. Also, publicly available databases are being launched to enhance interpretation of genomic sequence information, promoting mutual data sharing between expert consortia, professional organizations, health care providers, and patients. An inventory of the GWAS catalog in 2009 revealed that 88% of the GWAS associations are in either intergenic or intronic regions (28), regions of the genome we still understand little about, but to which GWAS has contributed by indicating regulatory sites (89). Moreover, the GWAS association signal in the radiographic vertebral fracture GWAS did not lie within a gene (59), and the same was true for some of the signals in the BMD and all-type of fracture GWAS (44). The Encyclopedia of DNA Elements (ENCODE) project, aiming to identify all functional elements in the human genome, has drastically enriched our comprehension about regions outside of the exome and showed that many GWAS SNPs overlap transcription-factor-occupied regions or DNase I hypersensitive sites and are particularly enriched in the segmentation classes associated with enhancers and transcription start sites (90). A striking finding is that obesity-associated noncoding sequences within the *FTO* locus are associated with expression of the homeobox gene *IRX3* at megabase distances, but not with expression of *FTO* itself; (91) this association seems to be driven by a topologically associated domain (TAD) structure encompassing the *FTO* and *IRXB* genes cluster (92). Such genomic explorations remain to be performed for osteoporosis-related traits.

### Pharmacogenomics

So far, therapies used to increase bone strength in individuals with osteoporosis are mainly based on antiresorptives (93). Bisphosphonates are the most widely used first-line because of their effectiveness, reasonable safety, and a low cost price (94). However, in practice, no single antiresorptive therapy is currently appropriate for all patients, as a subgroup of patients on anti-fracture medication responds suboptimally, e.g., small gain in bone mass or new fractures occur in spite of treatment, or negative side-effects such as osteonecrosis of the jaw or atypical femoral fractures (AFF) among others (95). To our knowledge no large-scale pharmacogenetic GWAS studies examining these phenomena in osteoporosis have been published to date, though initial case studies on the genetics of AFF and an accompanying systematic review have been published (96). In the future, results from pharmacogenomic studies may aid in assigning the most effective therapy to specific patient groups and it has been hypothesized that genetic biomarkers can be identified to pinpoint those patients most vulnerable to side-effects of certain agents. Nevertheless, because interaction studies tend to involve more parameters, up to four times as many subjects are needed (97); unless extremely large effects are in place, as we have witnessed for a few pharmacogenomic successes, such as anticoagulant dosing according to *VKORC1* haplotypes and HLA-B^*^5701 screening for the risk of hypersensitivity reaction to abacavir in HIV (98). Until now in genetic osteoporosis research, solely candidate gene studies have been performed investigating genetically-based variation in treatment response to raloxifene, teriparatide, and bisphosphonates (99). One of the reasons for this is that the coverage of pharmacogenomics variants was limited on GWAS genotyping platforms (100, 101), but this is improving with novel microarrays becoming available.

## Conclusion

GWAS is the study design necessary to further investigate the complex phenotypic and genetic architecture of osteoporotic fracture risk. Although fractures can be considered a complex trait, so far, the majority of susceptibility loci for fractures are also associated with bone mineral density. Hopefully, novel discoveries in the genetics of fracture risk will increasingly be translated clinical practice, with genotyping increasingly being successfully applied providing access to previously unknown information that may change the diagnostics and treatment of patients with bone diseases including osteoporosis with increased fracture risk in the future.

## Author Contributions

FK, KT, FR, and LO have written and revised the manuscript.

### Conflict of Interest Statement

The authors declare that the research was conducted in the absence of any commercial or financial relationships that could be construed as a potential conflict of interest. The reviewer RP declared a past co-authorship with the authors to the handling editor.
